# Balanced Options for Access and Benefit-Sharing: Stakeholder Insights on Provider Country Legislation

**DOI:** 10.3389/fpls.2019.01175

**Published:** 2019-10-01

**Authors:** Aysegul Sirakaya

**Affiliations:** ^1^Department of European, Public and International Law, Faculty of Law, Ghent University, Ghent, Belgium; ^2^ABS-int, Bruges, Belgium

**Keywords:** access and benefit-sharing, Nagoya protocol on access to genetic resources and the fair and equitable sharing of benefits, genetic resource, natural product research, utilization

## Abstract

The over-arching aim of the access and benefit-sharing (ABS) of genetic resources is to enable fair distribution of benefits between the users (such as universities and biotech companies) and providers (such as biodiversity rich countries) so as to both open the doors for innovation and create incentives for biodiversity conservation. Access to genetic resources is crucial for research related to conservation of plant genetic resources as well as R&D for agricultural products and evolved crops that can attain to the new weather conditions climate change brings. Therefore, access to genetic resources in general as well as benefit-sharing from that access is a key element for sustainable development in order to secure research as well as environmental sustainability and resource availability. ABS is currently a rapidly developing and evolving field that is shaped by each and every implementation of the Parties. This means that the national implementation of the Parties determine how ABS goals are realised and how ABS principles find form within regulatory mechanisms. These principles are found in international legal documents such as the Convention on Biological Diversity (CBD) as well as Nagoya Protocol. Additionally, decisions and guidelines drafted by the Conference of the Parties to the Convention on Biological Diversity shape these principles that are then to be fulfilled by the Parties when drafting their ABS laws by means of implementing regulatory mechanisms that comply with the international law. This article reviews 20 provider country’s ABS frameworks as well as one regional law with the aim of identifying the common regulatory mechanisms that find place in these legal texts. This descriptive approach is then followed by an empirical comparative analysis through semi-structured stakeholder interviews in order to identify the most beneficial regulatory mechanisms according to ABS experts that belong in four different stakeholder groups (provider countries, academic users, industrial users and collections)

## Introduction

Access and benefit-sharing (ABS) is a system under public international law that aims to fairly distribute benefits arising from genetic resources between the users of genetic resources (such as universities and biotech companies) and provider countries (regulatory authorities in biodiversity-rich countries). It is a system that finds its basic principles within the Convention on Biological Diversity (CBD) (United Nations Convention on Biological Diversity, 1992). These principles are further specified within the Nagoya Protocol on Access to Genetic Resources and the Fair and Equitable Sharing of Benefits Arising from their Utilization to the Convention on Biological Diversity (Nagoya Protocol, 2011). The CBD and the Nagoya Protocol, together with the decisions of the Parties thereof, establish the international ABS goals. These goals have been explored in a previous study by the author (Sirakaya, 2019).

ABS is a rapidly evolving field that is shaped by the implementation of the Parties to the CBD and the Nagoya Protocol. This means that the national implementation of these countries determines how ABS goals are realized and how ABS principles find form within regulatory mechanisms. These principles have to be implemented by the Parties when drafting their ABS laws by means of putting regulatory mechanisms in place which are in line with the international ABS goals. In other words, how provider countries regulate ABS directly shapes the way ABS principles are implemented.

This article reviews 20 of these national ABS laws and a regional ABS law implemented by provider countries throughout the world with the aim of describing the different types of regulatory mechanisms that provider countries use and providing examples of some of the countries that utilize them. This descriptive approach is then followed by an empirical comparative analysis through semistructured stakeholder interviews to identify the approach toward various mechanisms on access, benefit-sharing, and compliance of ABS experts that belong to four different stakeholder groups (provider countries, academic users, industrial users, and collections).

## Methodology

The methodology of this article consists of two stages. The first stage follows a descriptive approach in reviewing and explaining common regulatory mechanisms on ABS. Within this stage, a legal analysis of primary sources (national legislation, regulations, policies, and guidelines where applicable) within selected countries and regions is conducted to explore the commonalities and differences provider countries have in regulating ABS matters. Once identified, these regulatory mechanisms are briefly described and explained. The explanation is then followed by a comparative analysis of the selected countries’ and region’s related regulatory mechanisms regarding on access, benefit-sharing, and compliance.

The second stage follows an empirical approach to discover and analyze the stakeholder perception on these previously identified common regulatory mechanisms on ABS. A qualitative analysis in the form of semistructured interviews is conducted for acquiring stakeholder perceptions on these mechanisms, as well as for demonstrating the qualitative data on stakeholder perceptions on them.

The purpose of this article is not to fully describe these 21 legal documents in detail but rather identify common regulatory elements and subjects them to stakeholder interviews.

The choice of the countries subject to this review study is made based on the following criteria:

- Richness in biodiversity
- Diversity in terms of economic development
- Diversity in terms of maturity of the ABS framework
- Diversity in signatory status under the Nagoya Protocol^1^

The selection furthermore aims to demonstrate a worldwide approach as there is at least one country in each continent that has a state/region with an ABS framework. After applying these criteria, the following ABS frameworks have been selected for review: Andean Community, Australia, Brazil, Costa Rica, Dominican Republic, France, Ecuador, Ethiopia, India, Japan, Kenya, Malaysia, Namibia, Norway, Philippines, Republic of Korea, South Africa, Spain, Thailand, Uganda, and Vietnam.

Looking into regulatory issues related to ABS, the author has identified four key stakeholders: the government (as the provider), collections, academic users, and industrial users. These have been identified in line with Freeman’s definition of stakeholder, which is “any group or individual who is affected by or can affect the achievement of an organization’s objectives” (Freeman and Mcvea, 2001). These key stakeholders’ involvement in the regulatory processes is vital to form an ABS system that is effective and efficient and that attains the international ABS goals (Swiderska, 2001).

## Options in Access and Benefit-Sharing

With the aim of compiling the international ABS principles regulated under the CBD and the Nagoya Protocol with additions from various COP Decisions, the author has previously conducted the review of these international documents on ABS and compiled 11 ABS goals that are prescribed by these documents that are then to be fulfilled by the Parties through their national ABS frameworks. These goals found through the literature review conducted by Sirakaya (2019) are listed as follows:

1. Predictable conditions (Nagoya Protocol Preamble)
2. Legal certainty (Nagoya Protocol Article 6, COP Decision V/26, VII/19, VIII/4)
3. Transparency (Nagoya Protocol, COP Decision V/26)
4. Fairness and equity in negotiations (Nagoya Protocol, COP Decision V/26)
5. Sustainable use of biodiversity components (CBD Article 1, Nagoya Protocol Preamble, Article 8, Article 9, COP Decisions V/26 and VII/19)
6. Cost-effective measures (Nagoya Protocol Article 6, COP Decisions VII/19, VIII/4)
7. Scientific research based on genetic resources (CBD Article 15.6)
8. Strengthening the ability of indigenous people and local communities to benefit from the use of traditional knowledge (Nagoya Protocol Articles 5, 6, 7, 12, 21, 22, COP Decision V/26, VI/24)
9. Tech transfer and cooperation to build research and innovation capacity in developing countries (Nagoya Protocol, COP Decisions VIII/4, VII/19, VI/24, V/26)
10. Creating incentives to conserve biodiversity (CBD Article 11, COP Decision VI/24, Nagoya Protocol Preamble)
11. Innovative solutions for transboundary situations (Nagoya Protocol Preamble and Article 11)

In principle, provider countries’ national ABS frameworks should aim to attain these goals by means of implementing regulatory mechanisms that aid these goals’ principles. However, provider countries can significantly differ in their approaches when enacting provisions related to ABS of genetic resources. Furthermore, there exists no consensus regarding the state practice at the regulatory level. This is because some countries choose to enact specific laws on ABS, whereas some regulate ABS-related issues under framework legislation related to the environment or biodiversity or modify existing legislation to include ABS obligations. Nevertheless, similarities can be found with regard to the regulatory options that provider countries implement within the field of ABS. After analyzing 20 provider country approaches as well as one regional approach toward ABS, this section compiles the most commonly used regulatory options within the ABS frameworks of the provider countries into categories of regulatory mechanisms, such as material scope (what type of genetic resources are regulated), temporal scope (when can the ABS obligations be triggered), activity scope (which activities are regulated), geographical scope (within national laws, this comes up when regional competence or competence based on the type of genetic resource is divided), and other types of mechanisms found through the review of primary sources (i.e., country legislation on ABS), such as the requirement for an access permit (3.1.), requirement for a benefit-sharing agreement, standardized or negotiable conditions, types of monetary and non-monetary benefit-sharing found within the ABS frameworks (3.2.), and provisions on compliance and monitoring (3.3.) (**Table 1**).

**Table 1:** Commonly used regulatory options on access and benefit-sharing.

Commonly used regulatory options on ABS	Access	Scope	Material scope	*In situ* only
				*In situ* + *ex situ*
				Access to DSI
			Temporal scope	Sampling
				Utilization
				Access to a previously utilised genetic resource for new utilisation
			Utilization scope	Research
				Development
				R&D
		Pre-condition for access		Mandatory BSA
				Voluntary BSA
		Options for regulatory mechanisms		Permit
				Notification
		Standardisation		Standardised
				Case-by-case
	Benefit-sharing	Types	Monetary	Joint ventures
				Access fee
				Up-front payment
				License fee
				Royalties
				Salaries and funding
				Trust fund payment
			Non-monetary	Raw data
				Research results
				Capacity building
				Technology transfer
				Research directed towards priority needs of the provider country
				Food and livelihood security benefits
		Trigger		Access
				Utilisation
		Standardisation		Standardised
				Case-by-case
		Renegotiability		Renegotiable when the user and/or the intent changes
				Non-renegotiable
	Compliance	Sanctions		Administrative fines in any case of breach
				Criminal sanctions in any case of breach
				Administrative fines for light breach, criminal sanctions for severe breach

### Access

It should first be noted that neither the CBD nor the Nagoya Protocol defines access to genetic resources. Furthermore, the countries subject to this study either do not define access or define it in accordance with their understanding of access. Therefore, this study refrains from defining access and claiming either approach as the aim of this study is to point out the common elements in national regulatory options on ABS.

#### Options for Material Scope

- *In situ* access only: A permit or notification is only required when access happens within the geographical borders of the provider country. This is the classical access case foreseen by the CBD, where a researcher takes a sample of a genetic resource in a field/forest/nature reserve/public or private land (CBD.int, 2011).
- *In situ* and *ex situ* access: A permit or notification is required when access happens within the geographical borders of a provider country as well as through biodiversity biobanks, which are collections of biological samples that are held for preservation, research, and/or conservation purposes (e.g., genebanks, botanical gardens, natural history museums) (Shaw et al., 2014). Some frameworks (e.g., Brazil, 2015) enable the law to apply retroactively, by choosing to include the genetic resources accessed before their legislation came into force, within the scope of their ABS framework. This means that the material found in collections that were accessed before the enactment of the law would still require permit from the competent authority of the provider country. Other countries choose not to apply such retroactive provisions and only regulate the access that happens after the date of entry into force of their national ABS framework.
- *In situ*, *ex situ*, and access to digital sequence information (DSI): Both physical accesses, access through biobanks (i.e., collections) and access to DSI, are covered. DSI is not defined under the international legal sphere. It has been introduced to the Parties to the CBD and Nagoya Protocol during COP 13 UNEP (2016a, 2016b). Parties to the Convention are currently discussing the possible inclusion of access to or use of DSI within the scope of the CBD and the Nagoya Protocol (Laird and Wynberg, 2018). For instance, the Andean Community (1996) includes DSI within the scope of application.

#### Options for Mechanism to Trigger Access (Temporal Scope)

- Access for sampling: The access requirements are triggered before the material is sampled *in situ* or obtained from an *ex situ* source. In this case, the obligations come into place at the moment the user obtains the ability to perform R&D activities on the genetic resource. The obligations are triggered prior to performing these activities. Kenya (2006, 2013) follows this approach.
- Access for utilization: The access requirements are triggered after the user obtains the ability to perform R&D. The trigger here is not the physical access, which enables the user to conduct R&D activities, but rather the utilization activity itself. This is the approach taken by the Dominican Republic (2018) as it excludes the access of genetic resources by *ex situ* collections, solely for conservation purposes and not for utilization or third-party transfer. Brazil (2015) is another example of a country that does not place the trigger on access but rather on utilization.
- Access to a previously utilized genetic resource for new utilization: The requirements are triggered when a new utilization activity occurs to a genetic resource that was previously made available to the user. France (2016) explicitly mentions this in its legal framework.

It is crucial to keep in mind that having utilization as trigger for access does not necessarily mean that all of the genetic resources accessed prior to the enactment of the national laws are within the scope of ABS obligations.

#### Options for Utilization Scope

- Research: Access for research activities only. The access is only permitted for activities that do not involve any product/process development.
- Development: This refers to access for product/process development.
- Research and Development (R&D): Access for both research and development.

Here, it should be noted that the division between research and development is yet to be clarified. While the vast majority of the countries regulate R&D on genetic resources (e.g., Brazil, 2015), some countries (such as Ethiopia and Thailand) do not include this distinction or define these differences.

#### Benefit-Sharing Agreement as a Condition of Access

- Mandatory benefit-sharing agreement: This stipulates that a benefit-sharing agreement is to be signed between the provider and the user prior to access. This is the approach taken by France (2016), Thailand (2011), and Vietnam (2017).
- No mandatory benefit-sharing agreement, which means that there is no obligation to enter into a benefit-sharing agreement prior to access, yet this obligation may arise during different stages of R&D. Japan and the Republic of Korea do not require a benefit-sharing agreement prior to access.

#### Options for Regulatory Mechanisms

- Notification-based access: The user is to provide information regarding the modalities of access (defining material and temporal scope, persons/entities involved, transfer, intent, access and/or utilization) to the competent authority. However, in this case, the user can proceed with the activity without waiting for a response. Here, the notification would not qualify as a permit because a permit requires the applicant to wait for the authorization of the competent authority to commence its activities. TheRepublic of Korea (2017, 2018) is one of the countries that only requires notification to the competent authority regarding access.
- Permit-based access: Users who want to access a genetic resource must apply for access and wait for authorization prior to proceeding with their activities. This is referred to as the Prior Informed Consent (PIC) under Article 6 of the Nagoya Protocol. The majority of the countries have this as a regulatory mechanism for access.

#### Granting Authority

- Centralized, single institution, a one-stop-shop to go for the applicant: This is the case where only one authority or institution (e.g., a ministry, a research institute, a university, or an independent institution) has the competence for the entirety of the country and all types of genetic resources. The majority of the countries subject to this study have a centralized, single institution mandated to grant access to genetic resources.
- Several institutions mandated either according to the types of genetic resources or due to regional competence regarding genetic resources: In some cases, several regions may have their competence on the issues related to genetic resources from that region. This is especially the case for federal states. In addition, some states choose to have multiple competent authorities based on different types of genetic resources. For instance, the Ministry of Environment may be mandated to deal with genetic resources accessed from national parks, whereas the Ministry of Agriculture may be mandated for plant and animal genetic resources. Thailand (Thailand, Office of Natural Resources, Environmental Policy and Planning, 2014) is one of the countries that have several competent authorities depending on the type of genetic resource, whereas Spain (2007) has numerous competent authorities due to regional competence.

#### Standardized or Case by Case

- Standardized access conditions prescribed by law, regulations, and/or polices: Some countries choose to have PIC as a standard contract with predefined terms and conditions for access, often accessible through the annexes of the ABS law, the regulation, or online. Some countries, such as India, (2014), Spain (2007),South Africa (2008), and the Philippines (2005), on the other hand, specify the minimum content of the PIC within their ABS frameworks.
- Case-by-case conditions depending on the type of access and type of genetic resource: Some countries choose to have general principles within their regulatory framework on ABS, yet draft a unique, bilateral PIC document for each case. In some cases, the information required from the applicant is prescribed by law; in other cases, the applicant has to contact the competent authority to find out what documents are necessary for the case in hand. The majority of the countries tend to favor case-by-case negotiation in their national laws.

#### Mandatory Local Partner

- The user must apply for a permit with a local public or private partner, or the local partner has the responsibility to obtain and manage the permit. This local partner can be a university, a company, a nongovernmental organization, which, in theory, helps the user obtain legal certainty and takes part in the R&D activities on the genetic resources subject to the benefit-sharing agreement. The Philippines, for instance, is one of the countries that require a local partner.
- The user can apply for a permit without a local partner. This is the approach taken by France and Spain.

#### Facilitated Access for Non-Commercial Research

- Yes, non-commercial research is subject to favorable access conditions compared to commercial research. Favorable access conditions can be exemplified as simplified ABS systems for non-commercial research, where fewer documents are required from the applicant, a permit is given in a shorter time frame, or where benefit-sharing is done on non-monetary and/or voluntary basis. Australia (2012a), Spain, South Africa, and Thailand are some of the countries that have favorable access conditions for non-commercial research. Ecuador (2011), on the other hand, provides the option of framework contracts for non-commercial research.
- No, both commercial and non-commercial research is subject to the same conditions. Kenya follows this approach.

#### Options to Renegotiate ABS Contracts

- Renegotiation when the user changes: In the cases where a user transfers the genetic material to a subsequent user who is not a party to the contract between the user and a provider country, nor it is mentioned in the PIC that third-party transfer is allowed, the new user must obtain a new PIC either before or after it receives the material from the previous user.
- Renegotiation when the intent changes: In the cases where a user’s scope of activity regarding the genetic resource accessed from the provider country shifts from non-commercial to commercial research and where the PIC does not allow such research activity, the user is to then renegotiate the PIC conditions with the provider country before or after the commercial research activity begins.

For instance, Vietnam explicitly specifies both of these options.

### Benefit-Sharing

The explanation below categorizes benefit-sharing types as follows. Non-monetary and monetary: Based on whether the user pays benefits in monetary value or in actions.

- Mandatory and voluntary: Based on the government’s choice on making the benefit-sharing mandatory or voluntary for the user.

#### Non-monetary Benefit-Sharing

- Raw data: This type of data could pertain to the core information on genetic resource related to its phenotypic characteristics. Ethiopia (2006) foresees this type of benefit-sharing as a part of the obligations foreseen for the access permit holder.
- Sharing of research results: There is no indication on what research results mean. However, following the daily practice of research institutions, we could conclude that this would be the reports that describe the results of a research based on its methodology (Anderson, 2003). The user would then need to provide a report as part of the non-monetary benefit-sharing obligation. Some countries, such as Australia (2012b), and India, further explain which research results would be of interest to them.
- Capacity building: At the international level, capacity building (or capacity development) is defined as “any intervention or activity purposely designed to contribute to the development or strengthening of the capabilities of people, institutions and systems’’ (CBD.int, 2019; UN.org, 2019). Article 22/4 of the Nagoya Protocol includes the following categories within the scope of capacity building:

- Capacity to implement, and to comply with the obligations of, this Protocol;
- Capacity to negotiate mutually agreed terms;
- Capacity to develop, implement, and enforce domestic legislative, administrative, or policy measures on access and benefit-sharing; and
- Capacity of countries to develop their endogenous research capabilities to add value to their own genetic resources.

In addition, Article 22/5 further explains which actions would fall within the scope of these categories.

While some countries only mention capacity building as part of the non-monetary benefits (e.g., Australia; France (2017); and Kenya), some countries, such as South Africa (2008) and Uganda (2005), follow a more specific approach by including some of the capacity-building activities mentioned under Article 22/5 of the Nagoya Protocol.

- Technology transfer: Referring to Articles 15, 16, 18, and 19 of the CBD, Article 23 of the Nagoya Protocol obliges Parties to “undertake to promote and encourage access to technology by, and transfer of technology to, developing country Parties, in particular, the least developed countries and small island developing States among them, and Parties with economies in transition, in order to enable the development and strengthening of a sound and viable technological and scientific base for the attainment of the objectives of the Convention and this Protocol.” Some countries, such as Costa Rica (1998), take an approach where technology transfer is a state obligation embodied in the ABS framework, which means that when negotiating benefits, the state is obliged to seek out technology transfer options. Most countries, however, merely list technology transfer as a type of non-monetary benefit-sharing (e.g., Ethiopia, Kenya, and Vietnam). Some countries, like the Philippines (2005), include the terms and conditions of technology transfer in their standard ABS contracts. Ecuador’s ABS Framework obliges the parties to discuss technology transfer options during benefit-sharing negotiations.
- Research directed toward priority needs: The Bonn Guidelines on Access to Genetic Resources and Fair and Equitable Sharing of the Benefits Arising out of their Utilization (UNEP, 2002) includes this as a type of non-monetary benefit-sharing and exemplifies them as research related to health and food, taking into account domestic uses of genetic resources in provider countries. Some countries, such as India and Namibia (2017), list this non-monetary benefit-sharing option in their ABS frameworks. Australia refers to the Bonn Guidelines in their model benefit-sharing agreement.
- Food and livelihood security benefits: This category is also mentioned under the Bonn Guidelines. It is not defined what it covers nor what is entails. Yet, some countries (e.g., India, Uganda, and Vietnam) include this type of benefit within their list benefits.

#### Monetary Benefit-Sharing

- Joint Ventures: This Concept Is Not Defined Under the CBD or Its Nagoya Protocol, Yet the Organisation for Economic Co-operation and Development (OECD, 1993) Provides Us With the Following Definition: “A Joint Venture Is an Association of Firms or Individuals Formed to Undertake a Specific Business Project.” Most of the Countries Subject to This Study (E.G., Kenya, Namibia, Brazil, and India) Refer to Joint Venture as an Option for Monetary Benefit-Sharing and Do Not Oblige Parties to Enter Into Such Agreements.
- Access fee/fee *per* sample: This type of benefit-sharing occurs when the access obligations are triggered more as an administrative fee either *per* applicant or *per* sample. Today, some countries (e.g., Kenya and Namibia) only mention such an option as a type of monetary benefit-sharing within their ABS frameworks, whereas some countries (such as Malaysia, 2017; Vietnam) indicate the types of fees or sometimes the amount of fees (India; South Africa, 2012; the Philippines; Uganda, 2007) payable prior to access.
- Up-front payments: This concept was initiated in Costa Rica through the access-for-fee agreement between the National Biodiversity Institute (INBio) and the pharmaceutical company Merck (Sittenfeld and Gamez, 1993). The concept got furthermore enshrined in the Bonn Guidelines. This type of benefit-sharing occurs when the user pays not only for the access fee but also for the negotiated amount of benefit-sharing before utilization. Some of the countries, such as India, Namibia, Kenya, and Uganda, list up-front payments as a monetary benefit option within their ABS frameworks.
- License fee: There is no unified definition of license fees within the field of ABS; however, this type of payment is rather common in the field of intellectual property law. According to the World Intellectual Property Organization (WIPO), a licensing agreement is a partnership between an intellectual property rights owner (licensor) and another who is authorized to use such rights (licensee) in exchange for an agreed payment, which either comes across as royalties or license fees. Royalties are usage-based payments, whereas license fees often occur periodically (World Intellectual Property Organization, 2004). License fees have been used within the field of ABS on several cases. Most of the countries (e.g., Uganda, India) mention this as a type of monetary benefit option within their frameworks.
- Royalties: Royalties are usage-based payments made by the user of a genetic resource to the provider country. They are often agreed to as percentages of gross or net revenue. Percentages of royalty payments are foreseen both in the Brazilian and Costa Rica (2003) ABS systems. The Dominican Republic is one of the countries that mention this as a monetary benefit-sharing option.
- Salaries and research funding: The majority of the provider countries’ ABS frameworks (e.g., Namibia, Kenya, Uganda, and Ethiopia) have this included as a benefit-sharing option. Although some of them include this under non-monetary benefit-sharing, the performance of this act only requires the transfer of a monetary amount and thus is suited better as a monetary benefit option.
- Trust-fund payment: This is a type of benefit-sharing payment made directly to a fund foreseen under the national ABS framework. The current ABS Framework in South Africa foresees compulsory payment to be made to the Bioprospecting Trust Fund after concluding the benefit-sharing agreement.

Some countries, such as Vietnam, list various benefit-sharing options without differentiating between their monetary and non-monetary character. Here, it should also be noted that some of the benefit-sharing options that can be found under the Bonn Guidelines are not included here as they did not exist in the majority of the ABS frameworks within the countries that have been sampled in this study.

#### When Will Benefits be Transferred/Trigger for Benefit-Sharing

- At the point when access obligations have been triggered: This is the case for countries that oblige up-front payments, access fees, or fees *per* sample. The user is then to pay such fee prior to or within a specified period after the signing of the PIC.
- At the point of utilization: In this case, the user shares the negotiated or predetermined amount or performs the activity subject to the agreed non-monetary benefit during the R&D process.

India foresees both these options depending on the activity.

- At the point of commercialization: Most countries’ approach to benefit-sharing agreements is to negotiate a trigger that is tied to the commercialization of the product. Alternatively, some countries explicitly mention that they expect benefit-sharing at this stage. This option automatically comes across for countries that laid down obligations for users to pay license fees. Nevertheless, Brazil also follows this approach, despite having enacted the mechanism of royalty payments.
- At the point (or a specified period after) the user or provider receives first benefits from the utilization of genetic resources (GR): The user only benefits (in monetary terms) from the utilization only sometime after a product or a process developed through the utilization of genetic resources is finalized and released into the market. This fact is considered in Brazil as the Brazilian ABS Framework foresees the payment of 1% of annual net revenue.
- Payment tied to the application of or exploitation of intellectual property (IP) rights: This also comes across as milestone payments. Milestone payments are the type of payments in licensing agreements where the payment is triggered by an activity or occurrence of an event (Crama et al., 2009). In its model benefit-sharing agreement, Australia (2012), takes the exploitation of IP rights into account when establishing the trigger for benefit-sharing.

#### Exemption From Benefit-Sharing

- Exempting the user from benefit-sharing for certain types of use (e.g., no benefit-sharing needed when the utilization is directed at biodiversity conservation, food security): For example, India exempts collaborative research projects (subject to approval by the competent authority) as well as non-commercial utilization for publication purposes from benefit-sharing. In addition, Norway (2016) exempts private and non-commercial users from obtaining PIC and MAT for utilizing traditional knowledge associated with genetic resources.

#### Preset Conditions Versus Case-By-Case Negotiation

- Preset: Benefit-sharing conditions and triggers are set within the law, regulations, and/or policies. The user signs a standard contract drafted by the provider country. Australia’s ABS framework includes a model benefit-sharing agreement, whereas Indian ABS Guidelines specify the amount of monetary benefits to be shared in specific situations. On the other hand, the Brazilian ABS framework specifies the percentage of the benefit to be shared based on the annual net revenue obtained from a finished product or a process.
- Case-by-case negotiation: Benefit-sharing is subject to negotiation between the providers and users. The majority of the countries in this study (e.g., Kenya, South Africa, Thailand, France, Spain, and Costa Rica) have ABS frameworks that lead to case-by-case negotiation for access permits.

### Compliance

#### Sanctions

- Administrative fines in any case of breach
- Criminal sanctions in any case of breach

The Philippines foresees both administrative fines and criminal sanctions.

- Administrative fines for light breach, criminal sanctions for severe breach (misappropriation/intentional breach/repetitive noncompliance): This is the case for the Republic of Korea.

Other than sanctions, some countries have monitoring requirements to ensure compliance. For instance, Thailand obliges annual reporting throughout the R&D process, whereas Brazil requires notification prior to commercialization. However, since these options were not taken on by the majority of the countries subject to this comparative study, the options did not make it to the interviews.

## Interviews

The stakeholder survey conducted in an initial study by the author (Sirakaya, 2019)^2^ included a question on the participant’s availability for an in-depth interview regarding ABS options. Fifty-three of the respondents demonstrated their interest, and 20 ended up participating to the interview. The distribution of the participants among the stakeholder groups has proven to be rather homogeneous as five experts represented provider countries, six experts represented (public) collections, five represented industrial users (from various sectors, such as agriculture, pharmaceuticals, and industrial biotechnology), whereas four represented academic users (postdoctoral researchers and professors associated with various universities). Written informed consent forms were obtained from all of these experts.

The stakeholder interview has been designed in a semistructured manner. The questions on access and compliance asked stakeholders to rank the preference *per* regulatory option (**Table 2**) on a scale of 1 to 3, with 1 being the most favorable and 3 being the least favorable. The questions on benefit-sharing asked the stakeholders to rank the impact (from very positive to very negative) and burden (from burden to very heavy burden) of engaging in the given monetary or non-monetary option.

**Table 2:** Stakeholders’ preference on access.

	Material scope	Temporal scope	Utilization scope	Preconditions	Regulatory mechanisms	Granting authority	Standardization	Mandatory local partner	Facilitated access	Renegotiability
**Providers**	*In situ*, *ex situ*, and for DSI	Access for utilization	Development	Mandatory benefit-sharing agreement	Permit-based access	Centralized single institution	Case by case	Mandatory local partner	Facilitated access for non-commercial research	Renegotiable when user and intent change
**Academic Users**	*In situ* and *exsitu* access	Access for sampling and access for utilization	Development	No mandatory benefit-sharing agreement	Notification-based access	Centralized single institution	Case by case	No mandatory local partner	Facilitated access for non-commercial research	Renegotiable when user and intent change
**Industrial Users**	*In situ* and *exsitu* access	Access for utilization	Development	Mandatory benefit-sharing agreement	Notification-based access	Centralized single institution	Standardized and case by case1	No mandatory local partner	Facilitated access for non-commercial research	Renegotiable when user and intent change
**Collections**	*In situ* and *exsitu* access	Access for utilization	Research	No mandatory benefit-sharing agreement	Notification-based access	Centralized single institution	Standardized	No mandatory local partner	Facilitated access for non-commercial research	Renegotiable when user and intent change

^1^There was no consensus among industrial users regarding this option.

### Perceptions on Access

Question 1 was regarding the contact information of the stakeholders. Except for one participant, all of the interviewees representing provider countries were a part of the regulating body. Two of the provider country representatives were based in Africa, and the rest were scattered around the world. Participants representing collections were mostly based in the policy division of the collections they represented. All of the interviewees representing industrial users and academic users were based in either Europe or North America.

Due to the confidentiality concerns of the majority of the participants, the names of the interviewees will not be published.

Question 2 asked stakeholders to rank material scope options. The majority of the stakeholders opted for ABS frameworks to cover *in situ* and *ex situ* access, whereas the least favorable option stated by the majority of the stakeholders was *in situ* and *ex situ* access and access to DSI. It should however be noted here that the majority of the stakeholders answering as provider countries selected the inclusion of DSI as the most favorable option. Nevertheless, three of them did not do so. While one of them gave a middle-low score, the other two gave inclusion of DSI the lowest score. According to one of the latter, the reason for this is that this stakeholder could see that it would be hampering research even in the stakeholder’s own country.

Apart from two academic users, all of the user stakeholders found the inclusion of DSI the least favorable. One of them stated that it would not matter what is included in the scope as long as the regulatory requirements are not burdensome for whatever is covered in the ABS framework. One of the stakeholders who was against the inclusion of DSI suggested this to be handled in contracts rather than at the international level. This stakeholder argued that it is extremely challenging to define the limits of DSI in a unified manner. Another stakeholder from the collections argued that at the moment there is no way to track and trace DSI and, therefore, regulating it would be “a nightmare.”

The responses of industrial users and collections varied regarding their choice of the most favorable option. A stakeholder from the collections opted for “*in situ* access only” as it would be easier for collections to provide genetic resources. One of the industrial users that chose “*in situ* and *ex situ* access” as the most favorable option stated that in the sector the stakeholder is familiar with (plant breeding), *ex situ* access would be much more favorable as that sector tends to access *ex situ* rather than *in situ*. Responses from industrial users in different sectors (e.g., pharma) as well as collections and academic users also demonstrated different tendencies toward *in situ* versus *ex situ* access.

Question 3 asked stakeholders to rank temporal scope options. The majority of the stakeholders opted for “access for utilization,” which meant that they would prefer ABS obligations to be triggered at the moment of utilization of the genetic resource. “Access for sampling” took second place, whereas “access to a previously utilized genetic resource for new utilization” was the least favorable option. The preferences of the stakeholders in this question do not depend on the stakeholder group they belong in. For instance, where some stakeholders from the collections prefer the obligations to be triggered at the point of sampling (for it would bring legal certainty), others from the collections preferred that these obligations would be triggered at the point of utilization (as they believe that it may exclude most of the activities of collections). On the other hand, one of the academic users claimed that sampling in itself has no value and therefore should not be subject to ABS obligations.

Question 4 asked the preference of the stakeholders on what constitutes utilization. None of the options (research, development, R&D) had a significantly high preference rate as the choice of the stakeholders was scattered among all three options. Nonetheless, the option of utilization covering “R&D” got slightly higher votes than the others.

The vast majority of the stakeholders from collections opted for the ABS obligations to be triggered at the stage of research. The majority of the industrial users however found “development” to be the most favorable trigger. Neither the provider countries nor the academic users opted for an option more than the others.

Question 5 asked the stakeholders whether there should be a mandatory benefit-sharing agreement concluded prior to access. A slight majority (11 stakeholders or 61%) opted for mandatory benefit-sharing agreement rather than a no benefit-sharing agreement or voluntary benefit-sharing agreement prior to access. While all of the stakeholders representing provider countries opted for the mandatory benefit-sharing, some of the stakeholders that represent users also opted for this option, stating that having an agreement prior to access would define user obligations and thus help secure legal certainty. Industrial users had a higher preference rate toward mandatory benefit-sharing agreement compared to academic users or collections.

Question 6 on the choice between requiring a notification for access against permit for access received varied responses. The majority of the stakeholders representing provider countries opted for requiring a permit for access, stating it as the only way to ensure benefit-sharing. Some of the provider representatives however argued that notification could be accepted for either local researchers or non-commercial researchers as a whole. Some stakeholders representing users stated that permit is the only way to ensure legal certainty and to be certain that their access will not be challenged in the future. Some of the users, on the other hand, stated that the lengthy permit processes create burden for research and the bureaucracy that comes with the permit system in some cases can jeopardize public health in times of disease outbreaks. One of the collection representatives stated that notification is enough for monitoring the utilization of genetic resources, and the administrative burden that comes along with permit processes results in either missing out opportunities for research funding or no research at all.

Question 7 asked the stakeholders whether they would prefer one centralized competent authority or several authorities based on either regional competence or the type of genetic resource. The vast majority of the stakeholders stated that they would prefer a centralized, single authority for various reasons. First, for it would allow better monitoring of genetic resources; second, that it would ensure a standardized evaluation process; third, that it would minimize disputes and communication problems between the authorities; and last, it would bring down transaction costs for both parties (the costs for users to evaluate applications and/or monitor genetic resources as well as costs for users to obtain access to genetic resources).

The preference on question 8, which asked stakeholders their thoughts on standardized or case-by-case conditions on access, had the highest score supporting case-by-case conditions by slight majority. Nevertheless, all of the stakeholders that opted for the case-by-case conditions stated that the ideal situation would be standardized terms that have the flexibility to be adapted to a specific case. Some of the stakeholders who chose standardized conditions also stated that they would prefer a model contract that can be tweaked to meet the needs of the case, type of genetic resource, and type of access.

Question 9 asked stakeholders to pick between an ABS framework requiring a local partner prior to access and an ABS framework that either does not require such a condition or encourages it on a voluntary basis. The majority of the stakeholders opted for the latter. Not all provider country representatives were supportive of mandatory local partners. One of the provider country stakeholders expressed the need for capacity development for nominating local partners who can successfully handle such a task. This stakeholder further argued that most providers do not have such capacity. One of the stakeholders representing industrial users stated that small companies would also not be able to handle such a mandatory requirement. A stakeholder from collections argued that local partners are only beneficial for long-term projects, and such long-term partnerships can help develop capacity in provider countries, yet would only be a burden in short-term efforts.

Question 10, which asked whether the stakeholders would prefer an ABS framework that provides facilitated access to non-commercial research as well as other types of research addressed under Article 8 of the Nagoya Protocol, the vast majority chose the provision of such access. A collections representative defined facilitated access as fewer, simpler conditions where the non-commercial user can agree to share useful information related to the genetic resource with the provider country. An academic user representative stated that facilitated access would entail clear information on when and with whom the provider will need renegotiation in case of commercial exploitation. One of the provider country representatives stated that such facilitated access should especially be given to foreign researchers as they require additional assistance in accessing genetic resources compared to their local colleagues.

### Preferences on Benefit-Sharing

Questions 11–20 asked stakeholders how they perceive the impact and burden associated with several monetary and non-monetary benefits. These questions furthermore gathered insights from stakeholders regarding their preferences on the triggers and timing for benefit-sharing and the format and the mandatory nature of the benefits.

#### Non-monetary Benefits

##### Sharing Raw Data

The majority of the industrial users found sharing of raw data to be rather an ambiguous benefit-sharing option and a burdensome one. The majority furthermore exclaimed that the definition of raw data and what it entails are not clear. A way to encourage this is by giving the industry the choice of sharing it versus sharing other types of benefits.

Almost all of the interviewees from the collections were in favor of sharing raw data. The majority stated that generating data on genetic resources and making such data publicly available are highly beneficial for the collections and research dedicated to conserving biodiversity.

The vast majority of the interviewees from the provider countries stated that receiving raw data has a very positive impact. They however stated that there is some level of burden associated with it. Some of them stated that this burden comes from ensuring confidentiality to the data, and some of them stated that finding the right institution to share the data with to comprehend and make use of it is often challenging.

All of the academic users stated that sharing raw data would have a positive impact as academic researchers are also appreciative if the amount of publicly available raw data would increase. Some of them also argued that the term is rather ambiguous, and it should be standardized, or at least defined.

##### Sharing Research Results

The industrial users found sharing of research results to be a better option than sharing raw data. Yet, they stated that some burden is derived from inserting the research results into a usable format that is reader-friendly and is easy to disseminate.

The interviewees from the collections were all in favor of sharing research results as a type of benefit. They found it to have a very high positive impact also for their sector and stated that dissemination does not have much burden associated with it as it is one of their core activities.

Interviewees from provider countries found this option to be also highly beneficial for them, yet they stated that making sense of the results and being able to utilize them bear equal amounts of burden.

Academic users also expressed that sharing of research results is highly beneficial for them, and since it is their regular activity, such a benefit-sharing option would not be burdensome.

##### Capacity Building

The majority of the industrial users stated that this would have a relatively high positive impact, and the burden of executing such a benefit-sharing activity would not outweigh its impacts, whereas the majority of the interviewees from the collections stated that building capacity in provider countries has a highly positive impact both for the country and for the collections. They stated that capacity building helps establish more sustainable relationships with provider countries and also helps collections to ease into access procedures as mutual trust gets built.

This type of benefit-sharing is perceived by the majority of provider country participants to have a high positive impact. Some of the stakeholders argued that this would be the most important type of benefit-sharing as it would allow provider countries to valorize their own genetic resources, which they saw as the true meaning of the international ABS framework. However, they admitted that it would bear some limited amount of burden in ensuring that these activities would be received by the people who can utilize them.

While academic users stated that capacity building has a positive impact, they also argued that it has an equal amount of burden as the execution of capacity-building activities requires a relatively high amount of resources.

##### Technology Transfer

The interviewees representing the industrial users stated that this type of benefit-sharing has a high positive impact for them. Some perceived this to also have a positive impact for the provider country. Some argued that their scope of activities in conducting R&D with genetic resources results in a product or process that is a technology transfer activity in itself. Most of them also stated that technology transfer involves a limited to high amount of burden.

Likewise, the vast majority of the interviewees representing the collections found this to have a very positive impact. They added that compared to capacity building, technology transfer is a bit more burdensome.

Provider country participants stated that technology transfer is rather beneficial for them. However, learning and teaching how to make use of technology can sometimes be rather burdensome. Furthermore, the majority stated that not all technology they received was useful for them.

The majority of the academic users found technology transfer to have a lower amount of positive impacts than capacity building, stating that often they are not allowed by their research partners to engage in such an activity.

##### Research Directed Toward Priority Needs of the Provider Country

The vast majority of the industrial users were in favor of this benefit-sharing option. One of them underlined that this would be the best approach for his sector as benefits would directly return to the people who need them. Yet, the majority agreed on it as a heavy burden because making sure the research precisely helps the provider country would require a considerable amount of resources.

While the majority of the interviewees representing collections stated that this would generate positive impact, some of them expressed concerns for this option, stating that the collections are extremely constrained at the type of research they can engage in, and therefore, they would not always be able to secure funding for such benefit-sharing.

The majority of interviewees representing provider countries stipulated that this is rather a minimally burdensome type of benefit-sharing with a high positive impact. One of the interviewees stated that provider countries regularly look into research gaps, and identifying the ones that could be filled by benefit-sharing would constitute limited burden.

The vast majority of academic users stated that this benefit-sharing option fits within their scope of activities, and therefore, they would be able to maximize the positive impacts of conducting such research.

##### Food and Livelihood Security Benefits

This option was perceived as beneficial by the majority of the industrial users. While acknowledging the positive impacts of food and livelihood security benefits, some interviewees stipulated that this type of benefit-sharing often does not have a connection with the utilization of genetic resource itself and that many industrial users engage in this type of benefit-sharing regardless of having accessed genetic resources from that country.

The responses from interviewees representing collections were rather varied. Some of them claimed that this type of benefit-sharing does not fit within their sector’s scope of work, while others claimed that they have engaged in benefit-sharing activities that would be considered as food and livelihood security benefits. However, the majority argued that the burden of engaging in this option would outweigh the positive impacts.

Interviewees representing provider countries found this option to be the least impactful in terms of its positive effects for them, among other non-monetary benefits. One of the interviewees stated the reason for this as not being applicable to all of the regions or all of the provider countries. Nevertheless, the majority stated that the burden of receiving such a benefit would be minimal.

The interviewees representing academic users were not in favor of this option for their sector. While admitting it would still generate a limited amount of positive impact for them, it would also result in a heavy burden as this type of benefit-sharing is not something that they are used to see within their scope of activities.

#### Monetary Benefits

##### Joint Ventures

Almost all of the interviewees from industrial users stated that this would create a negative impact for their sector resulting also in very heavy burden. Some of the interviewees argued that, in some cases, a joint venture might work, but in any case, it should be a voluntary choice and not be imposed as a benefit-sharing clause.

The majority of the collections representatives stated that this option would constitute a very negative impact and even heavier burden. Some of them claimed that this type of benefit-sharing would only be relevant for applied research,^3^ yet they stated that the burden of keeping that joint venture functional would outweigh any positive impact.

While the majority of the provider country representatives stated that there could be potential positive impacts deriving from a joint venture, they also stated that the cost of establishing and sustaining such an initiative would outweigh all potential benefits.

The majority of the academic users perceived this to have a positive impact. One of the interviewees claimed that this would give researchers a chance to work with local strains alongside local researchers that have knowledge on them.

##### Access Fee per Sample

Almost all of the interviewees from industrial users stated that such a benefit-sharing option would create negative impacts for their sector, arguing that it is not a realistic approach as most sectors work with thousands of genetic resources at the same time. Some claimed that this would be impossible for small and medium enterprises and start-ups as the cost of access would start impacting the R&D process from the first step of the value chain onward.

All of the interviewees representing collections stated that this option would result in very negative impact and very heavy burden, arguing that collections do not have the budget to pay such a fee for each access.

While the overall result points out to a positive impact for provider countries, some interviewees argued that this would not be a satisfactory approach as they stated that the price paid for a sample would not constitute benefit-sharing.

Even though the overall result is a positive impact for academic users, some remained skeptical about this option.

##### Up-Front Payments

For similar reasons to access fee *per* sample, the majority of industrial users found this option to have negative impacts and heavy burden for their sector. They argued that benefit-sharing at the beginning of the activity would be a huge drawback as it discourages R&D.

All of the interviewees representing collections stated that up-front payments would result in very negative impact and very heavy burden. While agreeing with others, one of the interviewees stated that it might be interesting to share benefits up front if track and trace requirements would be removed by it and that the users would not have to worry about benefit-sharing at later stages.

The majority of provider country representatives perceived up-front payments to have a very positive impact in terms of being able to secure benefits from the starting point and being able to have less burden regarding enforcement and compliance.

The academic user interviewees did not have detailed opinions on this option. However, one remained skeptical, arguing that sharing monetary benefits from the get-go would negatively affect academic research.

##### License Fee

The responses from industrial user interviewees range from negative to very negative impacts as well as from heavy burden to very heavy burden. One of the interviewees argued that it is currently ambiguous what triggers sharing benefits as license fee. Another interviewee argued that license fees create a lot of administrative burden, which far outweigh positive impacts.

On the other hand, the vast majority of collections stated that this would not apply to them as collections do not engage in commercial activities with genetic resources. However, they claimed that if this would be applicable, it would create very negative impact and burden.

The responses from provider country representatives ranged from positive to very positive impacts, while burden was perceived to be limited. However, one of the interviewees opposed to it, arguing that imposing license fees would result in a lot of track and trace activities that create a heavy burden.

Although the majority of academic users perceived this benefit-sharing option to result in positive impacts and limited burden, they also refrained from clearly elaborating on the reason.

##### Royalties

The industrial user interviewees’ responses did not create a consensus on the impact of royalties for their sector. One of the interviewees argued that royalties would be burdensome in terms of the administrative work it requires.

The collections mostly stated that this type of benefit-sharing does not have an impact nor a burden for their sector as they do not engage in commercial research.

The majority of provider countries were in favor of this option as they stated that it would create a very positive impact. Yet, most of them argued that the administrative burden of establishing a system to organize receiving this benefit type would constitute a heavy burden. One of the interviewees stipulated that royalties acknowledge the provider country’s efforts to conserve biodiversity and create a good return on investment.

The majority of the academic user representatives did not have strong opinions against royalty payments. Some claimed that universities are prepared to execute such payment. Another one stated that collections may require a different type of benefit-sharing scheme, as this interviewee perceived collections’ work as already a type of non-monetary benefit-sharing in terms of biodiversity conservation.

##### Salaries and Research Funding

Among all of the other monetary benefit options, interviewees representing industrial users were in favor of this option the most. One of the interviewees argued that this type of benefit-sharing would go to the people who really need them, and another argued that this is a very useful option for strengthening the sector’s relationship with the provider country as they would be directly able to see the benefit that flows through the R&D on genetic resources.

Although the interviewees stated that the collections would have the least amount of burden in performing this benefit-sharing option, the majority still argued that collections do not have the capacity to provide such funding.

Provider countries did not favor this option as much as the latter two options. They stated that they would rather prefer funding for research, students, and capacity-building programs.

The majority of the academic users stated that they see this type of benefit-sharing (especially funding for PhD researchers) as one of their routine activities, and therefore, this would create minimal burden.

##### Trust Fund

The interviewees did not have a consensus on neither the impact nor the burden of this option. While some industrial users were indecisive about the effects it could have for their sector, some perceived it to be very beneficial.

Most of the interviewees from collections claimed that it would have no impact as they perceived that they would not be engaging in this type of benefit-sharing since they do not engage in commercial research. Some stated the benefit of a trust fund in terms of removing the burden of track and trace from both the user and the provider country.

Provider countries’ responses ranged from positive to very positive. According to one of the interviewees, a trust fund would help better organize benefit-sharing and transparency of transactions while enabling the provider country to reduce the cost of compliance checks.

Likewise, the majority of academic users were in favor of this option in terms of its potential to also simplify access for researchers.

##### Mandatory Versus Voluntary

Mandatory benefit-sharing was the dominant option for industrial users. One interviewee held that the industry would prefer mandatory benefit-sharing to ensure legal certainty. Another interviewee argued that the ideal option would be making benefit-sharing mandatory yet allowing users to pick between monetary and non-monetary.

For collections, the answers were two-fold. Half of the collections claimed that to ensure legal certainty, mandatory benefit-sharing is key while the other half argued that many of the benefits arising from collections work cannot be predicted in advance and may be delivered over decades; a flexible system is more suited.

All of the interviewees representing provider countries preferred mandatory benefit-sharing. However, one stated that it should be voluntary for local researchers. One interviewee claimed that his experience suggests voluntary benefit-sharing amounts to no benefit-sharing.

Only one of the academic users showed a tendency toward favoring mandatory benefit-sharing, arguing that provider countries will not be satisfied with a voluntary structure. The rest claimed that voluntary benefit-sharing would enable academic research to proceed.

##### Preset Versus Negotiated Conditions

For industrial users, the responses were two-fold. While the one half argued that small/medium enterprises (SMEs) would not be able to have resources to negotiate benefit-sharing agreements, the other half argued that everybody, including SMEs, has the means in its R&D budget to negotiate, and sometimes the flexibility provided by negotiation serves SMEs better. The former group furthermore argued that negotiating benefit-sharing each time an access happens bears too many transaction costs for both the user and the provider.

All of the interviewees representing collections were in favor of preset conditions as long as they would have some level of flexibility.

Provider countries’ responses were not in unison. While some claimed that preset conditions would be very beneficial in terms of reducing transaction costs, some stated that not all cases would benefit from such an approach.

The majority of academic users also opted for preset conditions. Some stated that they should be flexible enough to be adapted to the case in hand and should not be hindering R&D.

##### Trigger for Benefit-Sharing

The majority of industrial users preferred sharing benefits some time after the user benefits from utilization of genetic resources (e.g., after the product has been in the market for a year).

While some interviewees stated that this should be access for collections as collections do not engage in commercialization activities, some claimed that it is better to have the trigger as late as possible.

The vast majority of provider countries were in favor of the trigger to be at the point of access, stating that this is the only way to secure benefits and arguing that track and trace for provider countries is almost impossible.

Most of the academic users preferred the trigger to be commercialization, stating that academic research should not be bound by benefit-sharing obligations if there is no applied research that follows after.

### Perceptions on Compliance

Question 21 asked stakeholders their opinions on sanctions. The majority of the stakeholders preferred administrative fines for light breach, criminal sanctions for severe breach, while the least preferred option was criminal sanctions for all kinds of breach. Industrial users stated that they would prefer not to access genetic resources from countries that have criminal sanctions, especially for all kinds of breach. Academic users and collections stated the same regarding research activities. The majority of the provider countries emphasized the need to create proportionate sanctions.

## Conclusion

This study identified common regulatory options implemented by provider countries when regulating their ABS matters. Regarding access, the author identified 25 options on access and 6 non-monetary and 7 monetary benefit-sharing options that are common to the provider countries’ legislation subject to this study.

While describing the options, the research demonstrated some ambiguities regarding the definition and scope of some terms related to benefit-sharing. For instance, it was not possible to fully identify what constitutes sharing raw data, research results, or food and livelihood security benefits. Neither the international legal framework (CBD and the Nagoya Protocol) nor the COP Decisions prescribe what these benefit-sharing types exactly consist of. On the contrary, non-monetary benefit-sharing options, such as capacity building and technology transfer, are explained in detail at the international level. This comparative analysis furthermore noted that most of the ABS frameworks of the African countries subject to this study listed some benefit-sharing options within their legislation or annexed to the legal document without further describing them. It was noted that the majority of these options were identical to the options listed in COP Decision VI/24, also known as the Bonn Guidelines, which is the most detailed ABS guideline at the international level that got drafted before the Nagoya Protocol came into force. Although this seems to be a beneficial approach in terms of the national ABS frameworks’ compatibility with the international ABS principles, the interview with stakeholders further demonstrated that neither the regulators nor the users exactly know what actions some of these benefit-sharing options entail.

Moreover, the comparative analysis demonstrates that, apart from some (such as Brazil, India, South Africa, and the Philippines), the provider countries’ laws often do not expressly mention the trigger for benefit-sharing, meaning that the users would not be able to directly comprehend when they would need to share benefits. When this information is analyzed together with the data gathered from the provider country representatives during the interviews, we may think that this is perhaps because the majority of the provider countries opt for benefit-sharing at the point of access and that they presuppose the benefit-sharing will anyhow happen right after the PIC is granted and the mutually agreed terms (MAT) negotiated. This perception however needs to be tested in further detail.

The data related to access gathered during the stakeholder interviews lead to several conclusions. First, while the majority of the users do not favor the inclusion of DSI within the material scope, the majority of the provider countries do. This of course does not come as a surprise; however, an interesting point noted during the discussions is that even some of the provider country representatives admit that the inclusion would likely hamper the research, also for the local researchers in provider countries.

Regarding the activity that is included in the material scope, the participants agreed that the definition of utilization and the activities covered are yet to be clarified.

The data also demonstrate the ongoing lack of trust between users and provider countries. While provider countries want to subject access to a permit, users also want to be able to secure legal certainty from the get-go. In both cases, the stakeholders refer to previous biopiracy cases or allegations thereof as the underlying reason.

The responses from collections often demonstrate that they dissociate themselves from monetary benefit-sharing as there is a general perception among them that disentangles their scope of work with work that would require to share monetary benefits. They perceived that only commercial utilization would require monetary benefit-sharing.

It is also possible to see that both the users and provider countries still look for solutions to reduce transaction costs. The majority believes that multiple competent authorities in a country result in a lack of clarity and increase transaction costs, while most support facilitated access for basic research. However, some of the participants argued that the separation between basic and applied research is becoming increasingly complex. During the interviews, users in general repeatedly stated that complying with some ABS laws has proven to be especially difficult for non-commercial research and SMEs as the system is rather costly for them.

While some of the interviewees were not yet sure what to think of channelling benefits into a trust fund, the majority argued that such a benefit-sharing option would enable both the providers and the users to save on monitoring costs and that such a system could bring transparency into benefit-sharing. The stakeholders had a similar opinion regarding preset conditions for benefit-sharing. Although there is still no consensus on whether preset conditions would work, some stakeholders saw the benefit of them in terms of reducing transaction costs.

The results indicate a clash of opinions between provider countries and industrial users. Regarding benefit-sharing, where provider countries see a positive impact and minimal burden, industrial users often perceive negative impact and/or heavy burden. This is the case for sharing raw data and research results as well as paying access fee per sample and up-front payments. The clash also exists in providing salaries and research funding, where industrial users feel that it would create a very positive impact for them and for provider countries, yet the provider countries do not favor this option as much as the industrial users do. The trigger for benefit-sharing is another part where provider countries and the majority of the users disagreed. The providers stated that it would not be possible to retrieve benefits if they are not shared at the point of access, whereas the users in general stated that, in most cases, there is no benefit to share at the point of access as no utility is generated at that time.

Provider countries and industrial users do however feel the same way regarding some of the benefit-sharing options. For instance, both agree that capacity building is a very beneficial option with minimal burden. Likewise, they both agree with the fact that benefit-sharing should be mandatory. The majority of the industrial users expressed that the provider countries would not be satisfied with a voluntary benefit-sharing approach.

Apart from academic users, all of the stakeholders agreed that joint ventures, as a monetary benefit-sharing option, are heavily burdensome. It is perceived that the academic users perhaps do not associate a joint venture with establishing a joint corporate structure and carrying out the utilization of genetic resources under it (which is the way other stakeholders perceived joint ventures as such). For academic users, it seems that joint ventures indicate research collaborations with local institutions in the provider country.

While this study provides clarity to the perceptions of stakeholders on regulatory mechanisms commonly implemented by provider countries, further study is required both for finding a common ground between provider countries and industrial users and for a systematic analysis of each regulatory option’s capacity to attain the international ABS goals. After all, a balanced ABS system would be associated with trust while dissociated with ambiguity and complexity.

## Data Availability Statement

The datasets generated for this study are available on request to the corresponding author.

## Author Contributions

AS is the sole author of this manuscript.

## Funding

This study was supported by VLAIO/ IWT, Baekeland PhD Grant.

## Conflict of Interest

The author declares that the research was conducted in the absence of any commercial or financial relationships that could be construed as a potential conflict of interest.

## Footnotes

1 All of the selected countries are parties to the Convention on Biological Diversity, yet some of them have not yet ratified the Nagoya Protocol.

2 The survey has been sent to over 600 stakeholders including all of the national competent authorities of parties to the CBD, all of the national focal points, academic institutions, collections, and industrial users worldwide. The selection of the stakeholders is based on their function in their institution as well as their demonstrated interest in ABS (published articles, their position and expertise, attendance to conferences, workshops, or discussions related to ABS). Two hundred twenty responses were obtained.

3 Upon receiving this response, these interviewees were asked to clarify the difference between basic/fundamental research and applied research. All of them agreed that there is no clear line between where one ends and the other begins. One of them stipulated that it is not possible to realistically talk about fundamental research today, since even for a single research funding, scientists need to talk about valorization, innovation, and end result.

## References

[B1] Andean Community (1996). Decision No. 391 Establishing the Common Regime on Access to Genetic Resources.

[B2] Anderson P. V. (2003). Technical communication – A reader-centered approach. 5th ed. Boston: Heinle.

[B3] Australia (2012a). Explanatory guide: model benefit-sharing agreement.

[B4] Australia (2012b). Model access and benefit-sharing agreement between Australian Government and access party, Available at: http://www.environment.gov.au/system/files/pages/e3584028-d083-4aec-acdd-c0aa635a529f/files/commonwealth-and-access-party-model-benefit-sharing-agreement-2012.pdf.

[B5] Brazil (2015). Law 13,123, dated May 20, 2015 To regulate paragraph 1, item II and paragraph 4 of Article 225 of the Federal Constitution; Article 1, Article 8(j), Article 10(c), Article 15, and Article 16, items 3 and 4 of the Convention on Biological Diversity, enacted by Decree no 2,519, dated March 16, 1998; to provide for access to genetic heritage, for protection and access to associated traditional knowledge, and for benefit-sharing for conservation and sustainable use of biodiversity; to revoke Provisional Act no. 2,186-16, dated August 23, 2001; and for other purposes.

[B6] CBD.int. (2019). “Capacity Building”. https://www.cbd.int/cb/.

[B7] Cbd.int. (2011). ABS Information Kit - English [online] Available at: https://www.cbd.int/abs/information-kit-en/ [Accessed 23 May 2019].

[B8] Costa Rica (2003). Decreto N. 31514 del 3 de octubre de 2003 Normas Generales para el Acceso a los Elementos y Recursos Genéticos y Bioquímicos de la Biodiversidad.

[B9] Costa Rica (1998). Ley de Biodiversidad 7788.

[B10] Crama P. Reyck B. De Degraeve Z. (2009). Milestone Payments or Royalties? Contract Design for R&D Licensing. Operations Res. 56 (6), 1539–1552. 10.1287/opre.1080.0589

[B11] Dominican Republic (2018). Regulation of access to genetic resources and distribution of benefits (ABS).

[B12] Ecuador (2011). Reglamento Nacional al Régimen Común de Acceso a los Recursos Genéticos en aplicación a la Decisión N° 391 de la Comunidad Andina (Decreto Ejecutivo N° 905 de 3 de octubre de 2011).

[B13] Ethiopia (2006). Proclamation No. 482/2006 access to genetic resources and community knowledge and community rights.

[B14] France (2016). 3.1.LOI n° 2016-1087 du 8 août 2016 pour la reconquête de la biodiversité, de la nature et des paysages, les procédures d’accès et partage des avantages sur les ressources génétiques relevant de la souveraineté de l’Etat et les connaissances traditionnelles associées à ces ressources génétique.

[B15] France (2017). France Ministère de la Transition écologique et solidaire Arrêté du 13 septembre 2017 fixant le contrat type de partage des avantages découlant de l’utilisation de ressources génétiques prélevées sur le territoire national, mentionné à l’article R. 412-20 du code de l’environnement.

[B16] Freeman R. E. McVea J. (2001). A stakeholder approach to strategic management. Darden Business School Working Paper No. 01-02. Available at SSRN: https://ssrn.com/abstract=263511 or 10.2139/ssrn.263511

[B17] India (2014). Guidelines on access to biological resources and associated knowledge and benefits-sharing regulations.

[B18] Kenya (2006). The environmental management and co-ordination act (No. 8 of 1999) environmental management and co- ordination (conservation of biological diversity and resources, access to genetic resources and benefit-sharing) regulations.

[B19] Kenya (2013). An act of parliament to provide for the protection, conservation, sustainable use and management of wildlife in Kenya and for connected purposes [Act No. 47 of 2013, Act No. 19 of 2015, L.N. 105/2017.].

[B20] Laird S. A. Wynberg R. P. (2018). A fact finding and scoping study on digital sequence information on genetic resources in the context of the convention on biological diversity and Nagoya Protocol. 3, 1–77. Available at: www.cbd.int/abs/dsi-gr/ahteg.shtmlpeerreview.

[B21] Malaysia (2017). Access to biological resources and benefit-sharing bill.

[B22] Namibia (2017). Access to biological and genetic resources and associated traditional knowledge bill.

[B23] Nagoya Protocol (2011). Protocol on access to genetic resources and the fair and equitable sharing of benefits Arising from their utilization to the convention on biological diversity: text and annex. (Montréal: Secretariat of the Convention on Biological Diversity).

[B24] Norway (2016). Regulations relating to the protection of traditional knowledge associated with genetic material Regulation | Date: 2016-11-25.

[B25] OECD (1993). Glossary of industrial organisation economics and competition law, compiled by R. S. Khemani, and D. M. Shapiro, commissioned by the directorate for financial, fiscal and enterprise affairs, OECD.

[B26] Philippines (2005). Joint DENR·DA-PCSD·NCIP Administrative Order No. 1 Series of 2005 Guidelines for Bioprospecting Activities in the Philippines.

[B27] Republic of Korea (2017). The Act on Access and Utilization of Genetic Resources and Sharing of Benefits. Act. Genet. Resour.

[B28] Republic of Korea (2018). Ministry of Environment and Ministry of Foreign Affairs. A Guide to Act on Access to and Utilization of Genetic Resources and Benefit-Sharing in Republic of Korea.

[B29] Sirakaya A. (2019). “Mutually supportive ABS system for users and providers: stakeholder perception on ABS goals,” in press Special Issue on Sustainability and Law. (Wiley Journal on Sustainable Development).

[B30] Spain (2007). Ley 42/2007, de 13 de diciembre, del Patrimonio Natural y de la Biodiversidad.

[B31] Spain, Ministry of Ecological Transition (2019). Frequently asked questions regarding the application and authorization to access to Spanish genetic resources from wild taxa Available at: https://www.miteco.gob.es/en/biodiversidad/temas/recursos-geneticos/protocolo-de-nagoya/faq_autorizaciones.aspx Accessed 16.05.2019.

[B32] Sittenfeld A. Gamez R. (1993). Biodiversity by INBio. Biodiversity prospecting: using genetic resources for sustainable development. Washington: World Resources Institute, 69–98.

[B33] Shaw D. M. Elger B. S. Colledge F. (2014). What is a biobank? Differing definitions among biobank stakeholders. Clin. Genet. 85, 223–227. 10.1111/cge.12268 24001330

[B34] South Africa (2008). Regulations on Bio-prospecting, Access and Benefit-Sharing [Commencement of GN R138].

[B35] South Africa (2012). Department of environmental affairs, South Africa’s bioprospecting, access and benefit-sharing regulatory framework: guidelines for providers, users and regulators.

[B36] Sustainabledevelopment. Un.Org. (2019). Capacity-Building: Sustainable development knowledge platform. Sustainabledevelopment.Un.Org. https://sustainabledevelopment.un.org/topics/capacity-building.

[B37] Swiderska K. (2001). Stakeholder Participation in Policy on Access to Genetic Resources, Traditional Knowledge and Benefit-Sharing, Case Studies and Recommendations. International Institute for Environment and Development (IIED). https://pubs.iied.org/pdfs/9096IIED.pdf

[B38] Thailand (2011). National committee on conservation and utilization of biological diversity regulation on the criteria and methods of the access and benefit-sharing of biological resources B.E. 2554.

[B39] Thailand, Office of natural resources, environmental policy and planning (2014). access and Benefit-Sharing. Bangkok: Ministry of Natural Resources and Environment.

[B40] Uganda (2005). Statutory instruments 2005 No. 30. The national environment (access to genetic resources and benefit-sharing) regulations.

[B41] Uganda (2007). Guidelines for accessing genetic resources and benefit-sharing in Uganda.

[B42] UNEP (2002). UNEP/CBD/COP/DEC/6/24 Bonn Guidelines on Access to genetic resources and fair and equitable sharing of the benefits arising out of their utilization.

[B43] UNEP (2016a). CBD/COP/DEC/XIII/16 Digital sequence information on genetic resources.

[B44] UNEP (2016b). CBD/NP/MOP/DEC/2/14 Digital sequence information on genetic resources.

[B45] United Nations Convention on Biological Diversity (1992). 31ILM818, (entered into force Dec. 29, 1993).

[B46] Vietnam (2017). Decree on the management of access to genetic resources and fair and equitable sharing of benefits arising from their utilisation.

[B47] World Intellectual Property Organization (2004). Successful technology Licensing. WIPO (903), 1–52.

